# The Effect of Temperature on the Development of *Spodoptera frugiperda* (Lepidoptera: Noctuidae)

**DOI:** 10.3390/insects11040228

**Published:** 2020-04-07

**Authors:** Hannalene Du Plessis, Marie-Louise Schlemmer, Johnnie Van den Berg

**Affiliations:** Unit for Environmental Sciences and Management, IPM program, North-West University, Potchefstroom 2520, South Africa; marielouiseschlemmer1@gmail.com (M.-L.S.); johnnie.vandenberg@nwu.ac.za (J.V.d.B.)

**Keywords:** degree-days, development, fall armyworm, maize, pest management

## Abstract

The fall armyworm (*Spodoptera frugiperda*) is a pest of tropical origin which recently invaded Africa, the Far East and Australia. Temperature, therefore, plays an important role in its invasion biology, since this pest does not go into diapause. The aim of this study was to determine the development rate of *S. frugiperda* at different temperatures and to calculate the number of degree-days (°D) required for each stage to complete its development. This study was conducted at five different temperatures—18, 22, 26, 30 and 32 ± 1 °C. Larvae were reared individually in Petri dishes with sweetcorn kernels provided as food. The development rate of *S. frugiperda* increased linearly with increasing temperatures between 18 and 30 °C and larval survival was the highest between 26 and 30 °C. The optimal range for egg, larval and egg-to-adult development was between 26 and 30 °C. The optimum temperature with the fastest larval development rate and lowest mortality was at 30 °C. The pupal development period ranged between 7.82 and 30.68 days (32–18 °C). The minimum temperature threshold for egg and larva development was 13.01 and 12.12 °C, respectively, 13.06 °C for pupae and 12.57 °C for egg-to-adult development. Degree-day requirements for the development of the respective life cycle stages of *S. frugiperda* were 35.68 ± 0.22 for eggs, 204.60 ± 1.23 °D for larvae, 150.54 ± 0.93 °D for pupae and 391.61 ± 1.42 °D for egg-to-adult development.

## 1. Introduction

The fall armyworm, *Spodoptera frugiperda* (Lepidoptera: Noctuidae), was first reported in Africa by Goergen et al. [1]. A large area of South and Southeast Asia is highly suitable for year-round occurrence of the fall armyworm [2,3]. There are also important invasion routes from Africa into several countries in this region [3]. The pest spread to various areas in 2018 and 2019 and was reported for example from India [4], Thailand [5], Myanmar [6], China [7], Republic of Korea [8], Japan [9], the Philippines [10], Indonesia [11] and in recently also in Australia [12]. Pest biology, distribution and abundance are largely influenced by the relationship between temperature and the development rate [13]. Since the development of insects occurs within a specific temperature range, a change in temperature will, therefore, influence the development rate, the duration of the life-cycle and ultimately, survival [14]. An increase in ambient temperature to near the thermal optimum of insects cause an increase in their metabolism and, therefore, also their activity [15].

The thermal optimum is the temperature at which a species develops, reproduces and survives optimally [16]. Temperatures lower or higher than the optimum temperature lead to a decrease in the development rate [16]. Temperature influences the duration of each instar, as well as the number of instars that larvae go through before reaching the adult stage [17]. A faster development rate can be advantageous to insects since it results in less time spent in vulnerable stages during which they can be attacked by predators, parasitoids and entomopathogens [15]. The status of pest species is, therefore, affected by changes in climate and weather [18]. It is, therefore, important that the effect of temperature on the development of target insect species under the current changing climatic conditions is known, since this will contribute to risk analyses, forecasting and management strategies in order to minimize pest infestation levels [19]. 

Temperatures fluctuate in natural environments and affect insect population dynamics differently from conditions where insects are only exposed to constant temperatures. Insects develop faster under fluctuating temperatures when the maximum and minimum temperatures are within their optimal range of development [20]. However, studies of insect pest species at constant temperatures can be used to predict their seasonal and phenological development [21], pest population dynamics and timing of control strategies [22]. The objectives of this study were to determine the development rate of *S. frugiperda* at different constant temperatures, the number of degree-days (°D) required for each stage to complete development, as well as the degree-days required for overall egg-to-adult development.

## 2. Materials and Methods

### 2.1. Spodoptera Frugiperda Stock Colony

*Spodoptera frugiperda* larvae (F0 generation) were collected from maize fields at Delmas (26.1575° S, 28.5915° E), Mpumalanga province, South Africa. These larvae were reared in plastic containers (40 × 20 × 15 cm) with aerated lids and provided with maize leaves (PAN 6Q-121) as food. Food was replaced at three-day intervals. Larvae were reared separately from the 3rd instar onwards. This was done in small plastic containers (52 mm in height × 30 mm in diameter) with steel mesh-infused lids. Larvae were kept in a rearing room at 26 ± 1 °C, 65 ± 5% RH, and 14L:10D photoperiod until pupation. Pupae were sexed and kept in the same rearing room as the larvae. Pupae were observed daily until moths emerged. 

After the emergence of moths, single male–female pairs were confined to oviposition chambers in a rearing room maintained at the temperature and conditions ascribed above. The chambers and methods used are according to those described by Kruger et al. [23]. A plastic bottle (22 cm in height and 10 cm in diameter) was cut open at the top and filled with small crusher stones up to a height of 5 cm. One maize stem, 18 cm in length and 25–30 mm in diameter, with the whorl intact, was placed in an upright position inside the bottle. The stem was inserted 3–4 cm into the crusher stones to keep it upright. Water was added up to a level three-quarters of the height of the stones to provide humidity and to keep the plant parts fresh. The containers were covered with a fine gauze mesh to prevent the moths from escaping. The maize leaves and stems were replaced every day and inspected for egg batches.

### 2.2. Temperature-Dependent Egg Development

Egg batches from the stock colony were removed from maize plants within 12 hours of oviposition by cutting off the piece of leaf to which the egg batches were attached. Approximately 50 eggs were placed per small plastic container (52 mm in height and 30 mm in diameter) closed with a steel mesh-infused lid. These plastic containers were kept in a glass desiccator (150 mm in diameter) in which relative humidity (RH) was maintained at 70 ± 5% using a potassium hydroxide solution according to the method of Solomon [24]. The desiccators were kept at 18, 22, 26, 30 and 32 ± 1 °C in incubators with a 14L:10D photoperiod. The temperatures and RH in each desiccator were recorded at 30 minutes intervals using iButtons^®^ from ColdChain Thermo Dynamics (Fairbridge Technologies, Johannesburg, South Africa). The eggs were checked daily until they hatched and the number of days to hatching was recorded.

### 2.3. Temperature-Dependent Larval and Pupal Development

Eggs were collected from moths kept at 26 ± 1 °C, 65 ± 5% RH and a 14L:10D photoperiod and husbandry as described above. After hatching, neonate larvae (F_6_ generation) were transferred and kept individually in Petri dishes (9 cm in diameter) with sweetcorn kernels (cultivar NK603) in the soft dough stage as food. Larval and pupal development was studied under the same conditions of constant temperature and photoperiod as described above for egg development. The developmental times of the pre-pupal and pupal stages were combined and provided as the development time for pupae. 

The Petri dishes were checked daily for head capsules and exuviae. Head capsules and exuviae, if present, were removed from Petri dishes to avoid confusion when data on molting and survival were recorded. Food was replaced and the Petri dishes cleaned daily. Once the larvae pupated, the pupae were checked daily until the emergence of the moths. The number of days to emergence of moths was recorded. The temperature and RH inside the container at each temperature regime were recorded at 30 minute intervals using iButtons^®^ from ColdChain Thermo Dynamics (Fairbridge Technologies).

### 2.4. Data Analysis

The relationships between temperature (x) and the development rate (y) were determined by using a simple linear regression analysis. The lower threshold temperature (t) and the number of degree-days (k) required to complete the development of each of the stages, as well as their standard errors, were calculated using the equations provided by Campbell et al. [25]. The lower threshold temperature was estimated by setting y = 0 and solving x for the regression equation, y = a + bx, where y = 1/days, x = temperature, a = intercept and b = slope. The lower temperature threshold was calculated as t = −a/b and the thermal constant for development in number of degree-days (°D): k = 1/b. The standard error of means was calculated as the S.E. of t = $\bar{y}$/b √(s^2^/${N\bar{y}}^{2}$) + [S.E. of b/b]^2^ and standard error of the degrees-days as the S.E. of k = (S.E. of b)/b^2^. The mean number of degree-days (°D) needed for the development of the egg, larval and pupal stages were estimated using the equation of Jackson and Elliot [26]: °D = T (c − T_min_), where T is the number of days taken to complete development at a constant temperature (c) and T_min_ is the minimum temperature for development. The thermal constant was used and the mean number of °D required for the development of each life stage at the set constant temperatures were compared. Normality of the data on the effect of temperature on development was verified by means of the Shapiro–Wilk test, and for homogeneity, Levene’s test was used. Data were neither normally distributed nor homoscedastic and were, therefore, analyzed using the Kruskal–Wallis test followed by Duncan’s multiple range tests (*p* = 0.05). All statistical analyses were done using TIBCO Statistica™ 13.3 (Palo Alto, CA, USA) [27].

## 3. Results

The egg, larval and pupal development times of *S. frugiperda* were inversely related to temperature between 18 and 32 °C (Table 1). The development time of eggs (H (4, N = 139) = 138.00, *p* < 0.001)) and all larval instars differed significantly at temperatures between 18 and 32 °C (Table 1). [First instar (H (4, N = 139) = 96.41)) (second instar (H (4, N = 139) = 107.65, *p* < 0.001)); third instar (H (4, N = 139) = 117.79, *p* < 0.001)); fourth instar (H (4, N = 139) = 110.74, *p* < 0.001)); fifth instar (H (4, N = 139) = 111.05, *p* < 0.001)); sixth instar (H (4, N = 139) = 130.82, *p* < 0.001))]. The total duration of the larval development period also differed significantly at the respective temperatures (H (4, N = 139) = 128.52, *p* < 0.001)). The development times for all the above-mentioned stages were significantly longer at a constant temperature of 18 °C compared to 22, 26, 30 and 32 °C. Mortality of larvae at 18 °C was also very high (71%), indicating that a constant temperature of 18 °C was not suitable for the development of *S. frugiperda* larvae. The development time of eggs and all instars, except for sixth instar larvae, was similar at 26 and 30 °C. The development time of sixth instar larvae was significantly faster at 30 °C, compared to that at 26 °C, but did not differ significantly from the larval development time at 32 °C (Table 1). The development time for the total larval development period was also inversely related to temperature between 18 and 30 °C, with no significant difference in the development time at 30 and 32 °C (Table 1). The development time of larvae at 26 °C decreased by 28% compared to the development time at 22 °C. However, larvae developed significantly faster at 30 °C compared to 26 °C and there was no significant difference in the development time between 30 and 32 °C. The lowest larval mortality (4%) occurred at a constant temperature of 30 °C (Table 1). The optimum temperature range for larval development was, therefore, between 26 and 30 °C and the optimum temperature 30 °C. The development time of pupae was also inversely related to temperature from 18 to 32 °C. There was no significant difference in the pupal development time at 26 and 30 °C as well as at 30 and 32 °C (Table 1). 

**Figure 1:**
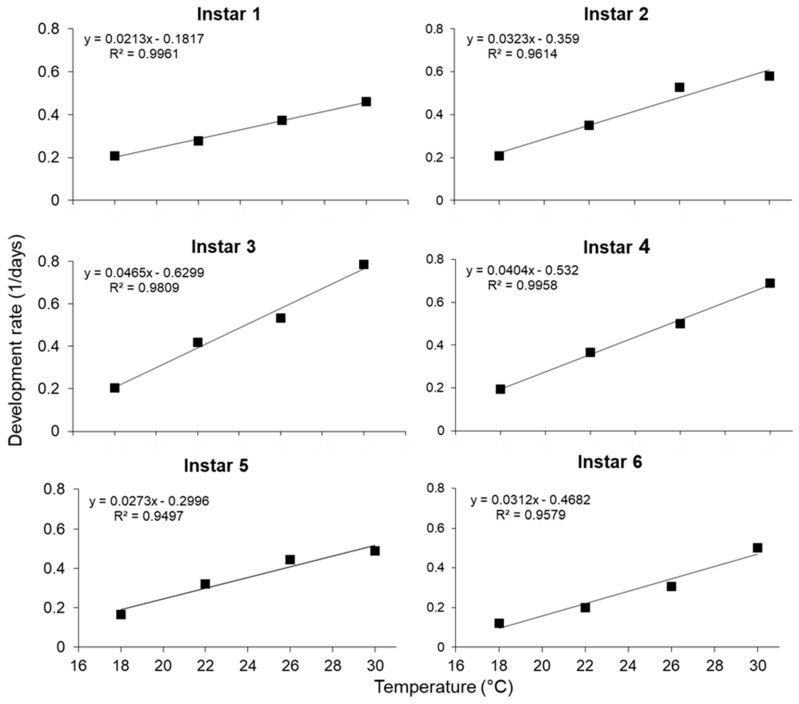
The relationships between *Spodoptera frugiperda* development rates and rearing temperature for larval instars one to six.

The relationship between temperature and the development rate of different larval instars of *S. frugiperda* is illustrated in Figure 1. Means were used to generate regression lines that described the relationships between tempature and the development rate (1/days). Linear regression equations describing these relationships and estimates of the lower temperature threshold (t) and the number of degree-days (°D) for each life stage are summarized in Table 2.

The thermal constant (k) for egg development was 35.73, and for the development of larvae and pupae, 202.67 and 147.06, respectively (Table 2). The number of degree-days required for egg-to-adult development was 391.01 °D (Table 3). For first instar larvae, the lower temperature threshold was calculated as 8.5 °C, which seems to be too low. A very high overall mortality rate of larvae (70%) occurred at 18 °C—most of which occurred during first-instar development. Therefore, using only the data on the development rate of 30% of the larvae that did survive to adulthood may explain this low estimated lower temperature threshold. Based on linear regression analysis of the development rate at all temperatures, a minimum temperature threshold of 13.01 °C was calculated for egg development and 12.12 °C for larval instars. The lower minimum threshold temperature for the larval stage was lower than that of the egg stage and eggs will, therefore, not develop and hatch at temperatures which are not suitable for larval development.

## 4. Discussion

The development rate of species within their favorable temperature range increases linearly, but the rate becomes nonlinear at unfavorable temperatures [28]. The egg development rate for *S. frugiperda* was similar at 30 and 32 °C in the current study, which is in contrast to the findings of Ali et al. [29] who reported a much wider favorable range of 17 to 38 °C. These authors found linearity in the egg development rate between 17 and 33 °C, and a decline in the egg development rate at 35.5 and 38 °C. Although viability was found to be high in this study with all eggs that hatched at temperatures ranging between 18 and 32 °C, development at a constant temperature of 18 °C was slow and the percentage of eggs that hatched was very low. Continuous low temperature, although above the lower thermal limit, will, therefore, slow down development and may reduce the population growth rate as a result of high mortality. The high viability of *S. frugiperda* eggs observed in this study is consistent with results from previous studies [30,31,32]. 

The development rate of *S. frugiperda* eggs, larvae and pupae increased linearly with temperatures increasing from 18 to 30 °C. Ali et al. [29] reported the development rate of larvae at temperatures ranging between 21 and 33 °C to increase linearly and, therefore, regarded the temperature range between 21 and 33 °C to be suitable for *S. frugiperda* development. In the present study, development at 32 °C was found not to be linear for egg and larval development, but linear for pupal development up to 32 °C. Since the optimal temperature range is a specific temperature range within the suitable range at which insect species can develop and reproduce [33], the optimal range for egg, larval and egg-to-adult development of *S. frugiperda* recorded in this study was between 26 and 32 °C. Larval survival was the highest between 26 and 30 °C and the optimum temperature with the fastest development and lowest mortality for larvae was 30 °C. *Spodoptera frugiperda* pupates at a depth of 2 to 8 cm below the soil surface under field conditions [34,35] and are, therefore, exposed to changes in daily temperatures [36]. Soil temperature can be very high (>30 °C) during summer months in South Africa, which also coincides with the maize production season and *S. frugiperda* infestation of maize. The ability of *S. frugiperda* pupae to survive and develop at high temperatures in the soil, therefore, provides an advantage for this pest in terms of its development and survival. The estimated lower development threshold of 13.01 ± 0.10 °C for eggs is near the thresholds estimated by Ali et al. [29] (12.69 ± 1.37 °C) and Hogg et al. [37] (13.4 °C), but lower than the 16.95 ± 1.35 °C threshold reported by Barfield et al. [38]. Busato et al. [31] reported different lower development thresholds (9.3 and 8.1 °C) for eggs of the corn strain of *S. frugiperda* collected in two geographically different areas in Brazil, and 8.1 °C for eggs of the rice strain, regardless of the area sampled. 

The larval development times of 34.39 days at 18 °C and 10.45 days at 32 °C in the current study was faster than those reported by Busato et al. [31], which were 41.9 days at 18 °C and 11.1 days at 32 °C. Larval mortality was the lowest between 26 and 30 °C, with 70% of larvae that died at 18 °C and 28% at 32 °C. Barfield et al. [38] also reported *S. frugiperda* larval mortality to be higher at 18 and 37 °C than at 26.7 °C. 

The duration of the overall development period (egg to adult) decreased from 71.44 at 18 °C to 20.27 days at 32 °C, which is faster than the 77.3 days at 18 °C and 21.0 days at 32 °C reported by Busato et al. [31]. This is also slightly slower than the total development period of 66.5 days at 18.3 °C and 18.3 days at 35.0 °C reported by Barfield et al. [38]. 

The development rate of successive generations can be determined by using the duration of the egg-to-adult period [25]. Degree-day requirements for *S. frugiperda* larval development were 202.67 ± 4.45 °D and 150.29 ± 2.79 °D for pupae, which is near the larval degree-day requirement of 197.60 ± 0.54 °D reported by Ali et al. [29], but differed from their 112.86 ± 9.25 °D reported for pupal development. Ali et al. [29] found degree-day requirements for pupae to be independent of larval diet. However, differences in the developmental times of *S. frugiperda* larvae reared on various artificial diets as well as plant material were reported [29,32,34]. Ali et al. [29] found that larvae reared on cotton leaves required 37% more degree-days to complete their development than larvae that were reared on an artificial diet or maize. Mortality of larvae on a cotton diet was also the highest. The 29.3 days required for completion of the life cycle determined in this study is in agreement the findings of Sparks [34] who reported the life cycle to be completed in 30 days at 25 °C. Pitre and Hogg [39] reared *S. frugiperda* on maize leaf tissue at 25 °C and the reported mean development times for instars 1 to 6 were 3.3, 1.7, 1.5, 1.5, 2.0 and 3.7 days compared to 3.0, 2.1, 2.0, 2.2, 2.3 and 3.4 days in this study where larvae were reared on sweetcorn kernels at 26 °C. Similar development periods in these two studies were, therefore, recorded, taking into account the difference in food and temperature. In addition, a difference between the development times of larvae from the rice and corn strains was also reported [31]. However, Nagoshi et al. [40] reported that the African *S. frugiperda* may represent a novel interstrain hybrid population and mentioned potentially uncertain behavioral characteristics of this population. 

The *Spodoptera frugiperda* pupal development time ranged between 7.82 and 30.70 days (32–18 °C), which is in a similar range (6.6–30.4 days at 32–18 °C) to that reported by Busato et al. [31]. Pupae kept at 15 and 35 °C by Simmons [36] took approximately 37.2 and 5.6 days, respectively, to complete the pupal stage. Luginbill [30] reported the pupal stage to be completed in 9–45 days depending on the temperature, and 17 days at mean temperatures of 22.3 to 26.6 °C. Capinera [35] reported the pre-pupa and pupal stages to range between two and four days and between eight to 30 days, respectively, depending on the temperature conditions. A mean pupal development time of 17.06 days was recorded at 22 °C, but only 11.43 days at 26 °C. *Spodoptera frugiperda* pupae can survive for long periods at 10 °C, for example, although no moths emerged from pupae, Wood et al. [41] reported survival up to 50 days and Simmons [36] observed survival up to 62 days. Pupae kept at 40 °C did not survive [36]. The lower threshold temperature for the pupal development in this study was 13.06 °C, which is similar to that of 13.30 °C reported by Vickery [42] in the southern USA. According to Simmons [36], 90% of the pupae eclosed at temperatures of 20, 25, 30 and 35 °C, but only 58 to 78 % eclosed at 15 °C, with no difference in percentage pupal eclosion between fluctuating and constant temperatures. 

Insects evade temperature extremes by means of behavioral avoidance, migration, diapause or in a highly altered physiological condition [43]. *Spodoptera frugiperda* do not diapause and, therefore, migrate to regions which have more favorable environmental conditions [30]. Although temperatures below 13 °C at the overwintering sites of *S. frugiperda* have been reported not allow survival of larvae and pupae [44], Sparks [34] estimated this minimum temperature for survival to be 10 °C. With regard to the northern hemisphere, survival of *S. frugiperda* occurs in the southern regions of Florida and Texas and during mild winters along the Gulf Coast [30,34,38]. *Spodoptera frugiperda* cannot, however, survive periods of extreme cold, as well as periods with mild cold and rainfall [30]. The eggs, pupae and adults can tolerate cold without developing cold hardiness, but not the larval stages [45]. Cold hardiness is a slow process that increases the tolerance of an insect to survive in cold environments [46,47]. 

Knowledge of the temperature thresholds of insects is important for predicting their potential distribution [48,49]. The respective developmental stages have specific temperature requirements, which is important for survival in specific environments [49]. The threshold temperatures determined in this study can be used to refine existing models estimating the areas suitable for crop cultivation to which *S. frugiperda* can migrate from its over wintering sites as well as areas with suitable environmental conditions for persistent occurrence. 

## 5. Conclusions

Temperature thresholds determined in this study, can be used as parameters to model areas that are suitable for predicting the potential distribution and permanent establishment of *S. frugiperda*. The optimal range for egg, larval and egg-to-adult development of *S. frugiperda* was between 26 and 30 °C. The minimum temperature threshold for egg development was 13.01 °C, and that for larvae and pupae was 12.12 and 13.06 °C, respectively. This indicates that *S. frugiperda* populations will not develop and persist in geographical regions where temperatures decrease to below these levels during winter months. 

## Figures and Tables

**Table 1:** The mean development time (days ± S.E.) of different life stages and larval mortality of *Spodoptera frugiperda* at constant temperatures. The range or number of days taken to complete a stage is shown in brackets.

Development Stage	Temperature (±1 °C)				
	18	22	26	30	32
Egg	6.38 ± 0.05 a	4.00 ± 0.00 b	3.00 ± 0.00 b	2.0 ± 0.0 c	2.0 ± 0.0 c
	(6–7)	−4	−3	−2	−2
Instar 1	4.94 ± 0.15 a	3.70 ± 0.10 b	2.90 ± 0.21 c	2.70 ± 0.08 c	2.70 ± 0.08 c
	(3–7)	(3–5)	(1–4)	(2–3)	(1–3)
Instar 2	4.52 ± 0.11 a	3.00 ± 0.12 b	2.14 ± 0.16 bc	1.90 ± 0.12 c	1.33 ± 0.08 c
	(3–7)	(2–5)	(1–3)	(1–3)	(1–2)
Instar 3	5.00 ± 0.12 a	2.48 ± 0.09 b	2.00 ± 0.10 bc	1.43 ± 0.11 cd	1.06 ± 0.04 d
	(4–6)	(2–3)	(1–3)	(1–2)	(1–2)
Instar 4	5.16 ± 0.09 a	2.85 ± 0.11 b	2.10 ± 0.10 bc	1.62 ± 0.11 c	1.52 ± 0.09 c
	(4–6)	(2–5)	(1–3)	(1–2)	(1–2)
Instar 5	6.16 ± 0.16 a	3.42 ± 0.15 b	2.33 ± 0.11 c	2.19 ± 0.13 c	1.79 ± 0.07 c
	(4–8)	(1–5)	(2–3)	(1–4)	(1–2)
Instar 6	8.61 ± 0.24 a	5.12 ± 0.16 b	3.38 ± 0.16 b	2.00 ± 0.00 c	2.06 ± 0.04 c
	(6–12)	(4–9)	(3–6)	−2	(2–3)
Larvae	34.39 ± 0.41 a	20.58 ± 015 b	14.86 ± 0.31 bc	11.38 ± 0.25 cd	10.45 ± 0.10 d
	(28–37)	(19–22)	(13–19)	(10–14)	(10–12)
Pupae	30.68 ± 0.28 a	17.06 ± 0.24 b	11.43 ± 0.22 bc	9.00 ± 0.12 cd	7.82 ± 0.10 d
	(28–34)	(14–20)	(10–13)	(8–10)	(7–9)
Egg to adult	71.44 ± 0.40 a	41.64 ± 0.32 b	29.29 ± 0.29 bc	22.38 ± 0.27 cd	20.27 ± 0.15 d
	(67–77)	(38–46)	(27–32)	(20–25)	(19–22)
Larval mortality (%)	71	37	15	4	28

Means within a row followed by the same letter are not significantly different (*p* < 0.05).

**Table 2:** Linear regression equations describing the relationship between the development rate (1/days) and temperature (18–30 °C) and the thermal requirements of different developmental stages of *Spodoptera frugiperda*.

Development Stage	Regression model	k ± S.E.	t ± S.E.	R^2^-Value
Eggs	y = 0.0280x − 0.3641	35.73 ± 0.31	13.01 ± 0.10	0.96
First instar	y = 0.0212x − 0.1801	47.14 ± 3.13	8.49 ± 0.97	0.69
Second instar	y = 0.0313x − 0.3315	31.98 ± 3.26	10.60 ± 1.28	0.48
Third instar	y = 0.0463x − 0.6242	21.58 ± 1.49	13.47 ± 0.67	0.67
Fourth instar	y = 0.0404x − 0.5293	24.78 ± 1.80	13.11 ± 0.73	0.65
Fifth instar	y = 0.0278x − 0.3113	36.03 ± 3.13	11.21 ± 1.04	0.56
Sixth instar	y = 0.0307x − 0.4560	32.57 ± 0.94	14.85 ± 0.24	0.92
Pupal stages	y = 0.0067x − 0.0869	150.29 ± 2.79	13.06 ± 0.19	0.97
Larval stage	y = 0.0049x − 0.0598	202.66 ± 4.45	12.12 ± 0.24	0.95
Egg- to-adult	y = 0.0026x − 0.0322	390.41 ± 4.83	12.57 ± 0.13	0.98

t = estimated lower temperature threshold; k = estimated thermal requirement in degree-days.

**Table 3:** The mean development time in days and degree-days (°D) for *Spodoptera frugiperda* at constant temperatures. Degree-days were calculated using the lower threshold temperature for development for each developmental stage (eggs = 13.01 °C, larvae = 12.12 °C, pupae = 13.06 °C and egg to adult = 12.57 °C).

Developmental Stage	Temperature (°C)	n	Development time (days ± S.E.)	Range	Mean number of °D ± S.E.
Egg	18	93	6.38 ± 0.05	6–7	31.84 ± 0.00
	22	130	4.00 ± 0.00	4	35.96 ± 0.00
	26	151	3.00 ± 0.00	3	38.97 ± 0.00
	30	128	2.00 ± 0.00	2	33.98 ± 0.00
	32	162	2.00 ± 0.00	2	37.98 ± 0.00
Mean					35.68 ± 0.22
Larvae	18	31	34.39 ± 0.41	28–37	202.20 ± 2.43
	22	33	20.58 ± 0.15	19–22	203.29 ± 1.49
	26	21	14.86 ± 0.31	13–19	206.22 ± 4.31
	30	21	11.38 ± 0.25	10–14	203.49 ± 4.53
	32	33	10.45 ± 0.10	10–12	207.84 ± 1.95
Mean					204.60 ± 1.23
Pupae	18	31	30.68 ± 0.28	28–34	151.55 ± 1.36
	22	33	17.06 ± 0.24	14–20	152.52 ± 2.16
	26	21	11.43 ± 0.22	10–13	147.89 ± 2.90
	30	21	9.00 ± 0.12	8–10	152.46 ± 2.03
	32	33	7.82 ± 0.10	7–9	148.08 ± 1.93
Mean					150.54 ± 0.93
Egg to adult	18	31	71.44 ± 0.40	67–77	387.94 ± 2.17
	22	33	41.64 ± 0.32	38–46	392.63 ± 2.98
	26	21	29.29 ± 0.29	27–32	393.31 ± 3.84
	30	21	22.38 ± 0.27	20–25	390.10 ± 4.73
	32	33	20.27 ± 0.15	19–22	393.90 ± 2.84
Mean					391.61 ± 1.42
